# Flavonoids as Aglycones in Retaining Glycosidase-Catalyzed Reactions: Prospects for Green Chemistry

**DOI:** 10.1021/acs.jafc.3c04389

**Published:** 2023-10-06

**Authors:** Michael Kotik, Natalia Kulik, Kateřina Valentová

**Affiliations:** Institute of Microbiology, Czech Academy of Sciences, Vídeňská 1083, CZ-14200 Prague 4, Czech Republic

**Keywords:** Glycoside hydrolase, Hydrolysis, Transglycosylation, Glycosyl donor, Glycosynthase, Glucosidase, Rutinosidase

## Abstract

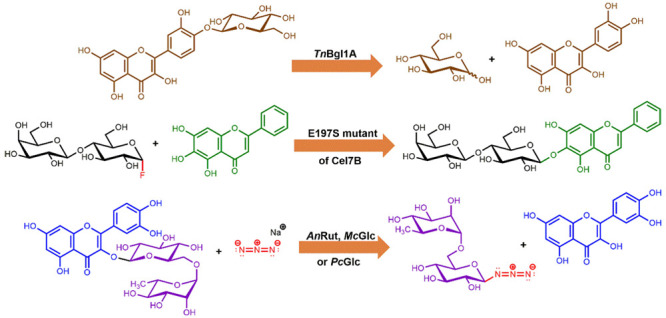

Flavonoids and their glycosides are abundant in many plant-based foods. The (de)glycosylation of flavonoids by retaining glycoside hydrolases has recently attracted much interest in basic and applied research, including the possibility of altering the glycosylation pattern of flavonoids. Research in this area is driven by significant differences in physicochemical, organoleptic, and bioactive properties between flavonoid aglycones and their glycosylated counterparts. While many flavonoid glycosides are present in nature at low levels, some occur in substantial quantities, making them readily available low-cost glycosyl donors for transglycosylations. Retaining glycosidases can be used to synthesize natural and novel glycosides, which serve as standards for bioactivity experiments and analyses, using flavonoid glycosides as glycosyl donors. Engineered glycosidases also prove valuable for the synthesis of flavonoid glycosides using chemically synthesized activated glycosyl donors. This review outlines the bioactivities of flavonoids and their glycosides and highlights the applications of retaining glycosidases in the context of flavonoid glycosides, acting as substrates, products, or glycosyl donors in deglycosylation or transglycosylation reactions.

## 1 Introduction

Flavonoid aglycones and their glycosylated counterparts are very abundant secondary metabolites in plants and fungi. They play an important role in nature, being involved in, e.g., plant defense mechanisms and plant-symbiont interactions. Flavonoid glycosides, which constitute a large part of the human diet, are also associated with beneficial health effects; therefore, they are of great importance in human nutrition. Their consumption appears to reduce risk factors for diabetes and cardiovascular and oncological diseases.^1^ In general, the glycosylation or deglycosylation of flavonoids has a far-reaching impact on their physicochemical and organoleptic properties and *in vivo* bioactivities. Considering all these facts, flavonoids and their glycosides have gained much importance in food technology and biological and biomedical research. In addition, certain natural flavonoid glycosides can be used as low-cost glycosyl donors for transglycosylation reactions catalyzed by retaining glycoside hydrolases. As discussed below, this class of biocatalysts has practical advantages over nature’s preferred glycosyltransferases in the glycosylation of aglycones. Importantly, enzymatic glycosylations are often viewed as a valuable, sustainable and simpler alternative to the synthetic chemistry approach with its weaknesses and shortcomings, such as waste production, use of hazardous reagents and petrochemical-based solvents, higher energy requirements, need for protective groups, formation of byproducts, lower overall yields, and low selectivity and atom economy.^2,3^ In the context of green chemistry, enzymes are biodegradable catalysts with high turnover numbers, rendering them highly efficient tools for synthesizing compounds in green processes. In particular, enzymes can be engineered to increase their stability and activity with artificial substrates and generate new products. In the context of flavonoid glycosides, there are three applications of retaining glycosidases and their engineered mutants: (1) deglycosylation in food processing and analysis, (2) the formation of flavonoid glycosides as products of transglycosylation reactions, and (3) the use of flavonoid glycosides as glycosyl donors. By focusing on these three topics, we highlight in this review the recent progress made in retaining glycosidases that accept flavonoids as aglycones. However, we do not consider whole-cell biotransformations in our review article. Moreover, we have excluded sucrose- and starch-dependent glycoside hydrolases because they have been recently reviewed elsewhere, with the exception of glycoside phosphorylases,^4^ which we have included in our review.

## 2 Flavonoids and Flavonoid Glycosides

**Figure 1:**
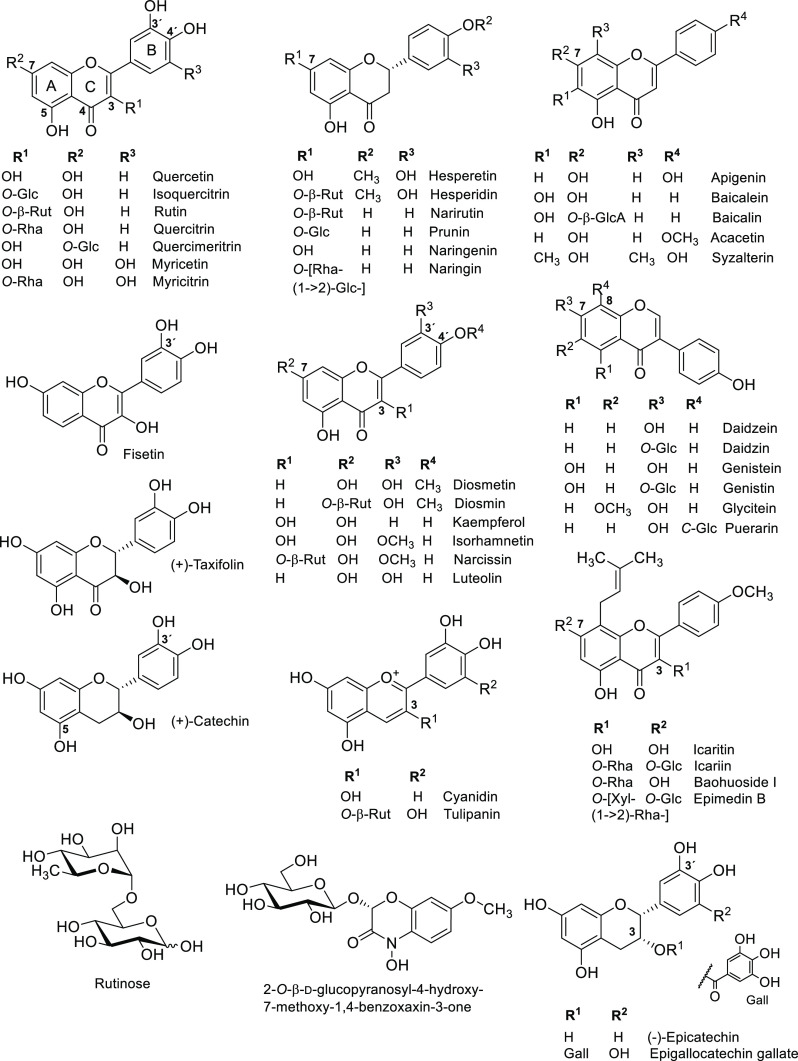
Structures of flavonoids discussed in this review. The structures of rutinose (6-*O*-α-l-rhamnopyranosyl-β-d-glucopyranose) and 2-*O*-β-d-glucopyranosyl-4-hydroxy-7-methoxy-1,4-benzoxaxin-3-one are also depicted. Gall, galloyl; Glc, β-d-glucosyl; GlcA, glucuronosyl; Rha, α-l-rhamnosyl; Rut, rutinosyl; Xyl, β-d-xylosyl.

Flavonoids are a large group of thousands of polyphenolic compounds with a common three-ring structure that includes two phenyl rings and one heterocyclic ring (Figure 1). They are ubiquitous secondary metabolites produced in plants and fungi.^5^ In plants, they are involved in various functions that are associated with color and pigmentation, resistance to drought conditions, increased salt and UV-light stress, the presence of heavy metals in the soil, defense against herbivorous insects, and inhibition of certain disease-causing bacterial and fungal organisms.^6−8^ For example, a high flavonoid content in tomato plants has been shown to confer resistance to the whitefly *Bemisia tabaci*, which feeds on these plants and can thus transmit plant viruses.^9^ On the other hand, high levels of flavonoids can harm beneficial organisms such as *Orius sauteri*, which are used for biological control of pests in tomato plants.^10^ Flavonoids, as cell wall components, are also involved in plant defense against fungal pathogens, as has been shown in several pathosystems.^11,12^ In addition, flavonoid glycosides and their aglycones play multiple roles in interactions between roots and microbial communities^13^ and may act as mediators in symbiotic interactions between nitrogen-fixing *Rhizobia* and legume roots.^7^

Most flavonoids in plants are glycosylated, linked to the carbohydrate moiety usually at position C-3 or C-7, forming *O*-glycosides (glucosides, rhamnosides, rutinosides, galactosides or arabinosides).^14^ Moreover, flavonoid *C*-glycosides and additional linking positions of the glycosyl moieties are also known.^15−17^ Besides glycosylation, other types of flavonoid derivatization exist. For instance, methylation of the flavonoid core structure 4′,5,7-trihydroxyflavone has been reported, such as in acacetin and syzalterin^18^ (Figure 1). Prenylation is another natural derivatization strategy, as found in, e.g., icaritin.^19^ Glycosylation of flavonoids, usually performed by stereospecific and regiospecific UDP-glycosyltransferases, can have several effects. It usually improves the solubility and stability of the flavonoid in aqueous solutions^20^ and can significantly alter the bioavailability of flavonoids. For example, certain isoflavone glycosides were suggested to be latent compounds that must be activated by deglycosylation to attract rhizobia in plant host-symbiont interactions.^13^ On the other hand, the glycosides of the flavonol quercetin are considered to be more bioavailable than the corresponding aglycone due to the higher solubility of the conjugates in the intestinal lumen. The literature suggests that the type of the sugar moiety attached and the type of glycosidic bond (*O*- or *C*-glycosides) has an impact on the bioavailability of flavonoids in the human gut.^21^ Deglycosylation of quercetin glycoconjugates is thought to be required prior to absorption, although there are conflicting reports, which found substantial amounts of intact isoquercitrin or rutin in rat plasma after administration of flavonoid-rich plant extracts.^22−25^ Deglycosylation is achieved either by a combination of brush border and cytosolic epithelial enzymes specific for the glucosyl moiety in the small intestine (lactase-phlorizin hydrolase, LPH, also known as lactase or cytosolic β-glucosidase) or by the action of rhamnosidases and rutinosidases (α-l-rhamnosyl-β-d-glucosidases) from the microbiota in the colon. Therefore, quercetin glucosides are predominantly taken up into enterocytes in the small intestine, whereas the aglycone from the corresponding rhamnosides and rutinosides is absorbed in the colon.^26,27^ After absorption, quercetin is methylated or converted into glucuronides or sulfates prior to entering the bloodstream.^26^ Importantly, the gut microbiota interacts with and transforms ingested flavonoids. This leads to the formation of new metabolites, which in turn modulate the microbial composition, such as increasing the number of bifidobacteria and lactobacilli, which has been shown to have positive health effects.^28,29^ Many flavonoids that are part of a balanced human diet have been shown to be involved in health benefits with antioxidant, anti-inflammatory,^27,30,31^ antidiabetic,^32^ antiviral, anticancer and protective activities,^33−35^ including prevention of cardiovascular diseases.^36^ Flavonoids act as antioxidants by virtue of their hydroxyl groups, which can scavenge reactive oxygen species (ROS) such as free hydroxyl radicals, which leads to a reduction in the effects of oxidative damage.^37^

In addition, flavonoids and their metabolites plausibly modulate redox signaling pathways and the expression of certain genes.^38^ Moreover, flavonoids and flavonoid-containing extracts have the potential to act as natural alternatives to synthetic pesticides.^18^ Several flavonoids have been found to inhibit α-glucosidase activities. Because α-glucosidases release d-glucose from sucrose and starch-derived oligosaccharides and are therefore associated with postprandial hyperglycemia, the administration of flavonoids may be a promising approach for the treatment of metabolic syndrome and diabetes.^39^

In food technology, the removal or deglycosylation of naringin, one of the main contributors to bitterness in orange and grapefruit juices, has been described as a treatment that improves juice quality because the released aglycone naringenin is perceived as less bitter.^40^ As an example, the deglycosylation of naringin, leading to the release of β-d-glucose, α-l-rhamnose and naringenin, was performed with an α-l-rhamnosidase and a β-d-glucosidase, which were coimmobilized onto magnetic silica-based chitosan microspheres.^41^ Similarly, so-called naringinase, i.e., an enzyme preparation that contains α-l-rhamnosidase and β-d-glucosidase activities, was immobilized onto graphene oxide for the production of prunin and naringenin from naringin with excellent process stability.^42^ Grapefruit pith is a rich source of naringin and narirutin and is a valuable byproduct of the fruit juice-producing industry. Treatment of grapefruit peel extract with a purified β-d-glucosidase from *Aspergillus niger* resulted in an increase in antioxidant activity and glucose levels, opening up the possibility of using the treated extract as a low-cost food supplement.^43^ Another potential application of specific β-glucosidases can be envisaged in the processing of tea for the hydrolysis of flavonoid glycosides, thereby improving its aroma.^44^ Because deglycosylation of flavonoids is often associated with higher antioxidant activities,^45^ the production of the flavanones hesperetin and naringenin from citrus peel extracts can be considered a practical and sustainable process in the context of food supplements.^46^

Covalent coimmobilization of an α-l-rhamnosidase and a β-d-glucosidase from the extremophile *Halothemotrix orenii* on functionalized agarose enabled the continuous hydrolysis of rutin and hesperidin to the corresponding aglycones using a packed bed reactor design.^47^ The reaction was performed in a biphasic mixture containing 5 mg mL^–1^ of flavonoid glycoside in buffer and TMO. After passage through the flow bioreactor, the aglycones were isolated from the organic phase by evaporation in very high yields.

Another interesting application of β-glucosidases is emerging in the deglycosylation of *Epimedium* flavonoids in Herba Epimedii, an herbal medicine consisting of dried leaves of various plants of the genus *Epimedium*. It contains a mixture of various glycosylated flavonoids, of which icariin is a major constituent. Because fully or partially deglycosylated *Epimedium* flavonoids, such as icaritin or baohuoside I, which occur in nature only in very small amounts, have shown better pharmacological effects than icariin, enzymatic treatment of *Epimedium* flavonoids improves their medicinal potential.^48^ The deglycosylation of *Epimedium* flavonoids, which contain glucosyl, rhamnosyl and xylosyl units, to the high-value aglycone icaritin, using the combined action of a β-d-glucosidase and an α-l-rhamnosidase—either in free or immobilized form—, has been the subject of a number of publications.^49−52^ Deglycosylation of all *Epimedium* flavonoids, some of which contain diglycosyl residues, was facilitated by the use of multifunctional glucosidases with β-xylosidase activity or rhamnosidases with the ability to handle both inner and outer rhamnosyl residues. Even a multifunctional glycosidase from *Aspergillus* sp. was reported that was able to cleave all glucosyl, rhamnosyl and xylosyl residues from the main *Epimedium* flavonoids icariin and epimedin B, resulting in the formation of the aglycone icaritin.^53^

Another example of the development of foods enriched in bioavailable (deglycosylated) flavonoids is the reported construction of lactic acid bacteria and *Bifidobacterium* strains with higher efficiency of deglycosylation due to the presence of a glucosidase-encoding gene from *Lactobacillus mucosae*. The strains were tested for the deglycosylation of, e.g., daidzin, genistin, and quercetin- and kaempferol-containing glycosides in a soy beverage fortified with a lignin-containing extract of flaxseed.^54^ Similarly, in view of a possible application in the deglycosylation of isoflavones in soy products, β-glucosidases from *Aspergillus oryzae* were presented on the surface of *Saccharomyces cerevisiae* cells, providing a biocatalyst for the production of the aglycones daidzein, genistein and glycitein from extracts containing isoflavone glycosides.^55^ Due to their health benefits, the use of flavonoid glycosides and their aglycones as nutraceuticals or dietary supplements is becoming increasingly popular. For instance, diosmin, isolated from citrus fruit peels, is used to treat chronic venous disorders.^56,57^ Another potential application of flavonoids and their glycosides as antiaging agents is emerging in cosmetics, as shown for hesperidin and its hydrolysis products hesperetin, rutinose and α-l-rhamnose.^58,59^ As important secondary metabolites, flavonoid diglycosides, such as rutin, hesperidin or naringin, are found in high concentrations in specific parts of plants, such as seedpods, fruit peels and pith.^60−62^ For instance, high rutin contents of up to 8% and 15–20% (based on dry weight) have been reported in the pericarp of fava d’anta (*Dimorphandra mollis*) fruits and *Sophora japonica* buds, respectively.^60^ Another example is Tartary buckwheat bran, which contains 3.0–8.6 g rutin per 100 g of dry weight.^63^ A high naringin content of up to ∼1.7 g per 100 g has been determined in grapefruit pith.^40^ In addition, the peel of sweet oranges represents a rich source of hesperidin, occurring at 1.5–2.0% fresh weight.^64^ The flavanone glycoside contents in the edible fractions of citrus fruits are lower. For instance, the hesperidin content in lemons has been found in the range of 2–142 mg per 100 g of fruit. Grapefruit contains up to 50–75 mg of naringin per 100 g of fruit or juice.^40,61^ Consequently, waste products and byproducts from the processing of citrus fruits and certain crops contain large amounts of flavonoid glycosides that can be used as a prospective starting material for enzyme-based reactions. In particular, the diglycosides rutin, hesperidin and naringin represent both very convenient and inexpensive substrates for hydrolytic reactions and glycosyl donors for transglycosylation reactions with flavonoid-accepting β-glucosidases (after trimming with α-l-rhamnosidases) or β-rutinosidases.

On the other hand, many flavonoid glycosides are present only in minute amounts in their natural environment. To explore their role in nature, a facile and high-yielding synthesis of these compounds, which serve as standards, reference samples, and probes in biological tests and analyses, is required. Undoubtedly, the synthesis of flavonoid glycosides is becoming increasingly important in biochemical, biomedical, and pharmaceutical research. As discussed later in the section on transglycosylations, flavonoid-specific β-glucosidases and rutinosidases can be very valuable catalysts for the synthesis of novel glycosides using low-cost flavonoid glycosyl donors. However, this approach using retaining glycoside hydrolases is in fierce competition with both the chemical synthesis of glycosides and enzymatic reactions using specific synthesizing glycosyltransferases.

The present review aims to summarize recent progress in reactions with flavonoid glycosides as substrates, products, or glycosyl donors using purified retaining glycosidases. An important prerequisite for successful enzymatic processes is a thorough characterization of the catalysts, especially their substrate, glycosyl donor, and acceptor specificities.

## 3 Enzymatic Hydrolysis of Flavonoid Glycosides

### 3.1 β-Glucosidases

β-Glucosidases (EC 3.2.1.21) catalyze the hydrolysis of glucoconjugates, releasing the terminal β-d-glucose from the nonreducing end. Based on their substrate specificities, β-d-glucosidases are classified into aryl-glucosidases, which hydrolyze aryl-glucosides, disaccharide-specific cellobiases, and β-d-glucosidases with broad specificity.^65^ β-Glucosidases are very abundant in microorganisms and plants. Their role in nature has been elucidated in a number of cases, such as their involvement in fungal catabolism of plant-derived flavonoid glycosides as the sole source of carbon and energy,^66^ or fungal degradation of plant cell walls.^67^ In addition, β-glucosidases were found to hydrolyze plant-based phenolic glycosides in midguts of larvae of *Papilio glaucus* butterflies (Eastern tiger swallowtail),^68^ which is thought to be important for the feeding ecology of this insect. In addition, it has been suggested that the hydrolytic action of β-glucosidases releases the biologically active isoflavone aglycones daidzein and genistein from the latent isoflavone-containing glycosylated forms in the root exudates of *Glycine max* (soybean) in response to the action of symbiotic and pathogenic microorganisms.^13^ There are a surprising number of putative β-glucosidases in plant genomes, highlighting their central importance. For example, 40 and 48 putative GH1 β-glucosidase-encoding sequences were discovered in the genomes of rice^69^ and *Arabidopsis*,^70^ respectively. β-Glucosidases, together with UDP-glycosyltransferases, are involved in various processes in response to abiotic and oxidative stresses in plants via (de)glycosylation of specialized metabolites, such as flavonoids with antioxidant properties or phytohormones.^71,72^ According to their amino acid sequence similarities, β-glucosidases have been classified into a number of glycoside hydrolase families in the CAZy (carbohydrate-active enzymes) database. They are currently represented in ten families: GH1, GH2, GH3, GH5, GH30, GH39, GH116, GH131, GH175 and GH180. The first six and the last one include enzymes whose catalytic domains have (β/α)_8_-barrel folds, while GH116 and GH131 consist of enzymes with catalytic domains having (α/α)_6_-barrel and β-jelly roll folds, respectively. They are all expected to have a retaining catalytic mechanism with potential transglycosylation activities, which are the basis for the synthesis of new glucosides in the presence of a donor glucoside and an acceptor other than water.

In the last two decades, more than a dozen β-glucosidases with activities toward flavonoid glucosides have been described. A large proportion of these were recombinant enzymes with relaxed specificities, predominantly originating from bacteria, especially bifidobacteria^73^ and lactobacilli, but also from plants. Sequence data suggest that multiple copies of β-glucosidase-encoding genes exist per bifidobacterial genome. It can be envisioned that probiotic bifidobacterial strains, as part of our diet, may help to enhance the health benefits of certain foods by releasing aglycones from ingested glucosides via their multiple β-glucosidase activities. A few enzymes have been shown to efficiently hydrolyze not only flavonoid glucosides but also disaccharides, such as cellobiose (4-*O*-β-d-glucopyranosyl-d-glucopyranose) and gentiobiose (6-*O*-β-d-glucopyranosyl-d-glucopyranose), to the monosaccharide d-glucose. Given the broad substrate spectrum of most of these enzymes, it seems difficult to precisely determine their role *in vivo*. Nevertheless, the frequent occurrence of daidzin and genistin as good substrates in the below-mentioned survey of β-glucosidases is striking and in line with their known abundance in legumes^74^ (e.g., in the context of defense responses in soybean^75^) and suggests their importance for microbial degraders of plant (legume) material.

In the following, we have sorted the β-glucosidases with activities toward flavonoid glucosides according to their reported substrate specificities.

**a) Broad specificity.** Several β-glucosidases have been shown to have very relaxed substrate specificities, accepting 3-*O*-, 7-*O*- or 4′-*O*-linked flavonoid glucosides and sometimes disaccharides (Table 1). Such broad substrate specificity suggests a high potential of these enzymes for the deglycosylation of various phytochemicals. In microorganisms, they are likely involved in the degradation of plant-based flavonoid glycosides.

**Table 1:** β-Glucosidases with Relaxed Substrate Specificities toward Flavonoid β-d-Glucosides

GH family	enzyme(s)	species^a^	flavonoid glucoside substrate^b^	relative activity [%]	remarks	ref
–	–	*Citrus sinensis* (p)	hesperetin 7-*O*-linked	199^c^	main enzymatic activity toward the disaccharides cellobiose, laminaribiose and gentiobiose	(155)
			naringenin 7-*O*-linked	138^c^		

–	G_II_	*Penicillium decumbens*	daidzein 7-*O*-linked (1.6)	1^c^	in addition, hydrolysis activity toward cellobiose and gentiobiose	(156)
			apigenin 7-*O*-linked (1.5)	1^c^		

GH1	Bbg572	*Bifidobacterium lactis*	daidzein 7-*O*-linked	–	activity toward isoflavanones, quercetin glucosides, and glucosyl-based disaccharides with β-1→2, β-1→3, β-1→4, and β-1→6 glycosidic bonds; hydrolysis products were identified by TLC	(157)
			genistein 7*-O*-linked			
			glycitein 7*-O*-linked			
			quercetin 3*-O*-linked			
			quercetin 4′*-O*-linked			
			quercetin 7*-O*-linked			

GH1	Llbglu1	*Leucaena leucocephala* (p; legume family)	genistein 7*-O*-linked	–	–	(158)
			naringenin 7-*O*-linked			
			apigenin 7-*O*-linked			
			genistein 4′-*O*-linked			

GH1	NfBGL595 wild-type enzyme	*Neosartorya fischeri*	apigenin 7-*O*-linked (747)	2^b^	–	(107)
			genistein 7*-O*-linked (1670)	5^b^		
			quercetin 3*-O*-linked (759)	2^b^		

GH1	*Tn*Bgl1A wild-type enzyme	*Thermotoga neapolitana*	daidzein 7-*O*-linked	100	a mutagenesis study was performed based on the crystal structure of *Tn*Bgl1A (PDB 5IDI; see text)	(106)
			genistein 7*-O*-linked	100		
			kaempferol 7-*O*-linked	100		
			quercetin 4′-*O*-linked	100		
			quercetin 3,4′-*O*-diglucoside	82		
			quercetin 3*-O*-linked (101)	73 (5^c^)		
			kaempferol 3*-O*-linked	87		
			isorhamnetin 3*-O*-linked	93		

GH1	–	*Thermobifida fusca*	daidzein 7-*O*-linked	100	in addition, activity toward cellobiose and cyanidin 3-*O*-β-d-glucoside	(159)
			genistein 7*-O*-linked	62		

GH3	*S*BGL	*Novosphingobium* sp.	daidzein 7-*O*-linked (33300)	–	in addition, hydrolysis activity toward cellobiose, gentiobiose, and amygdalin	(126)
			genistein 7*-O*-linked (19200)			

GH3	GluD_His_ and GluE_His_	*Bifidobacterium pseudocatenulatum*	genistein 7*-O*-linked	–	in addition, hydrolysis activity toward cellobiose and gentiobiose; hydrolysis products were identified by TLC	(160)
			daidzein 7-*O*-linked			

GH5	EXG1, SPR1 and YIR007W	*Saccharomyces cerevisiae*	daidzein 7-*O*-linked	–	EXG1: PDB 1H4P	(161)
			genistein 7*-O*-linked			
			naringenin 7*-O*-linked			
			kaempferol 7*-O*-linked			
			luteolin 7*-O*- and 4′-*O*-linked			
			quercetin 4′-*O*-linked			

a (p): plant.

b Where available, the corresponding *k*_cat_/*K*_M_ values [s^–1^ mM^–1^] are indicated in parentheses.

c Relative to the activity toward *p*NP β-d-glucopyranoside (100%).

**(b) Specificity for 7-***O***-linked flavonoid glycosides.** β-Glucosidases with less broad specificity are summarized in Table 2. In these cases, however, it is often unclear whether the limited substrate specificity is genuine or simply due to the use of a limited variety of substrates. Regarding the use of β-glucosidases for the deglycosylation of flavonoid glycosides, enzymes with large inhibition constants for the reaction product glucose are of particular interest (Table 2). It is worth noting that GmICHG from *Glycine max* (soybean) exhibited much higher *k*_cat_ values for 6″-*O*-malonylated daidzin and genistin (daidzein and genistein 7-*O*-(6″-malonylglucoside)) compared with the corresponding nonmalonylated compounds (Table 2). In this context, it should be noted that isoflavonoids, such as genistin and daidzin, are predominantly malonylated in soybean, which is the most common source of these compounds in the human diet.^76^ Abundant isoflavone malonyltransferases are responsible for the malonylation of these glucosides in soybean plants; however, for the time being, the biological role of malonylation appears to be unclear. Regarding the application of β-glucosidases for the hydrolysis of 7-*O*-linked flavonoid glycosides, the very high specificity constants of the thermostable *Pyrococcus furiosus* enzyme are striking due to the exceptionally high reaction temperature of 95 °C used. Even at the more practical temperature of 65 °C, the authors reported a high specificity constant of 5000 s^–1^ mM^–1^ for the hydrolysis of genistin.^77^

**Table 2:** β-Glucosidases with Activities toward 7-*O*-β-d-linked Flavonoid Glucosides

GH family	enzyme	species^a^	flavonoid glucoside substrate^b^	relative activity [%]	remarks	ref
GH1	Hbglu	*Hevea brasiliensis* (p)	genistin	100^c^	–	(162)
			glycitein 7-*O*-linked	96		
			daidzin	41		

GH1	Bgl1269	microbial metagenome	daidzin	100^d^	hydrolysis of isoflavone glycosides in soybean flour extract; *K*_i_ (glucose) of 4.3 M	(163)
			genistin	97		

GH1	GmICHG	*Glycine max* (soybean) (p; legume family)	genistin (400)	9	–	(13)
			daidzin (900)	17		
			genistin 6″-*O*-malonate (3900)	100		

GH1	–	*Pyrococcus furiosus*	genistin (12100)	100^c^^,^^e^	malonylated substrates were also accepted	(77)
			daidzin (4480)	37		
			glycitein 7-*O*-linked (1840)	15		

GH3	GluA_His_ and GluB_His_	*Bifidobacterium pseudocatenulatum*	genistin	–	hydrolysis products were identified by TLC	(160)
			daidzin			

–	ICHG	*Pseudomonas* sp.	genistin (443)	100	–	(164)
			daidzin (366)	97		
			apigenin 7-*O*-linked (170)	94		
			genistin 6″-*O*-malonate (56)	88		
			daidzin 6″-*O*-malonate (42)	84		

–	ICHG	*Glycine max* (soybean) roots (p; legume family)	genistin 7*-O*-linked (13)	16^c^	no activity with rutin or isoquercitrin	(165)
			daidzin 7-*O*-linked (34)	42		
			genistin 6″-*O*-malonate (81)	100		
			daidzin 6″-*O*-malonate (36)	44		

–	ICHG	*Cyamopsis tetragonoloba* (guar seeds) (p; legume family)	genistin	–	inactive toward rutin, naringin, and hesperidin	(166)
			daidzin			

a (p): plant.

b Where available, the corresponding *k*_cat_/*K*_M_ values [s^–1^ mM^–1^] are indicated in parentheses.

c Based on *v*_max_/*K*_M_.

d Based on hydrolysis productivity [mM h^–1^].

e Activity data determined at 95 °C.

### 3.2 β-Rutinosidases

Glycosidases that hydrolyze the glycosidic bond between the rutinosyl moiety of flavonoid rutinosides and the aglycone to produce rutinose (6-*O*-α-l-rhamnopyranosyl-β-d-glucopyranose) (Figure 1) have been characterized from fungi^78,79^ and a bacterial species.^80^ β-Rutinosidases are also found in plants, as demonstrated for *Sophora japonica* and Tartary buckwheat (*Fagopyrumtataricum*), which is a popular medicinal herb used in traditional Chinese medicine preparations and functional foods.^81−84^ Characterization of various β-rutinosidases revealed a fluid transition between narrow and broad substrate specificities. While certain rutinosidases appear to have a rather strict specificity for diglycosides,^80^ others accept both flavonoid glucosides and rutinosides with different preferences.^85−87^ To date, microbial β-rutinosidases have been found in three GH families. (1) *Acremonium* sp. served as the source of the only GH3 family rutinosidase (αRβG II) with hydrolytic activity toward 3-*O*-β-rutinosides (rutin, narcissin and tulipanin), 7-*O*-β-rutinosides (hesperidin and diosmin) and isoquercitrin.^88^ (2) The only bacterial enzyme from *Actinoplanesmissouriensis* showed hydrolytic activity with hesperidin and hesperidin chalcone and was classified as a GH55 member with a deduced inverting mechanism.^80^ (3) The other microbial rutinosidases were of fungal origin and found to be members of GH5_23 with broad substrate specificities.^89^ Activity data and the presence of N-terminal signal sequences indicated that most fungal β-rutinosidases are extracellular enzymes with typically acidic pH optima. A remarkable example of a β-rutinosidase with a low optimal pH of 2.2 was purified from *Penicilliumrugulosum*.^90^ The extracellular enzyme was found to hydrolyze rutin and isoquercitrin with almost equal activity. No activity was detected toward hesperidin.

Taking advantage of sequence similarity with *An*Rut,^91^ a corresponding gene was identified in the genome of *Aspergillus oryzae*.^92^ The recombinant enzyme *Ao*Rut was expressed in *Komagataella phaffii*(93) cultures and showed high specificity constants of 1.3–2.5 × 10^3^ s^–1^ mM^–1^ with a variety of rutinosides and glucosides, e.g., narirutin, rutin, isoquercitrin, prunin, and hesperidin.^86^ Thus, this broad-specificity enzyme was highly active for both 3-*O*- and 7-*O*-linked flavonoids.

An archaebacterial β-glucosidase with a very low rutinosidase activity was used for the hydrolysis of rutin (10 mM) at 95 °C.^94^ The gene encoding the enzyme was from the *Pyrococcus furiosus* genome. The authors reported a remarkably high thermal stability of the enzyme with a half-life of 101 h at 95 °C. Compared with the *k*_cat_/*K*_M_ of 303 s^–1^ mM^–1^ for isoquercitrin, the specificity constants for quercitrin and rutin were shown to be substantially lower by factors of 130 and 4000, respectively.

Because the low solubility of flavonoid glycosides is considered a major obstacle for economic large-scale hydrolysis reactions, cosolvents such as dimethyl sulfoxide have been used to dissolve flavonoid substrates. As a more environmentally friendly alternative, with low toxicity and good compatibility with enzyme-based reactions, deep eutectic solvents have been investigated as solubilizing agents for glycosylated flavonoid substrates.^95^ Using β-rutinosidase-mediated hydrolysis of hesperidin as a model reaction, mixtures of choline chloride–glycerol or choline chloride–ethylene glycol (30–40% in buffer, v/v) gave the most promising results in terms of enzyme activity. However, the authors used only a low concentration of 1.8 mM hesperidin in their biotransformation reactions.

Enzymatic hydrolytic reactions with flavonoid glycosides are usually carried out at low substrate concentrations in the presence of cosolvents to increase the solubility of the substrate, which has been determined to be 125 mg L^–1^ for rutin in water.^96^ However, cosolvents in the reaction mixture may affect enzyme stability and product isolation in subsequent purification steps. As an alternative, hydrolysis reactions catalyzed by *An*Rut were investigated in the absence of cosolvents with rutin concentrations of up to 300 g L^–1^ (∼0.5 M).^97^ Interestingly, under the reaction conditions of a slurry or paste containing predominantly undissolved rutin, the enzyme was shown to be active and produced quercetin, which precipitated as microscopic crystalline particles, recovered by simple filtration of the reaction mixture. Because no cosolvents or toxic chemicals were used, the process was environmentally friendly. Moreover, water-soluble rutinose was obtained from the filtrate as a valuable byproduct. Presumably, the enzymatic reaction took place in the saturated solution surrounding the undissolved rutin particles in the suspension. This “solid-state” enzymatic conversion is easily scalable and potentially applicable to other natural products.

Process productivities were reported for the enzymatic hydrolysis of hesperidin and rutin using β-rutinosidases of the GH5_23 subfamily. αRβG I was covalently immobilized on glyoxyl-activated agarose, resulting in a catalyst that was repeatedly used in 2-h conversions in the presence of 0.52 mM hesperidin and 10% (v/v) dimethyl sulfoxide at 60 °C. The catalyst could be reused for 15 cycles without significant loss of activity; a substrate conversion rate of 65% and a productivity of 3 μmol (g immobilized catalyst · h)^−1^ per cycle was obtained corresponding to 2 mmol (g enzyme · h)^−1^.^98^ Complete hydrolysis of mostly undissolved rutin at 185 g L^–1^ (0.3 M) in the absence of cosolvents was reported for two purified recombinant β-rutinosidases with productivities of 357 and 149 mmol (g enzyme · h)^−1^ calculated for rutin conversions of 20% and 70%, respectively.^85^ Because both rutin and the product quercetin were barely soluble in the reaction mixture, the reaction conditions remained heterogeneous throughout the conversion.

## 4 Enzyme–Ligand Interactions

**Figure 2:**
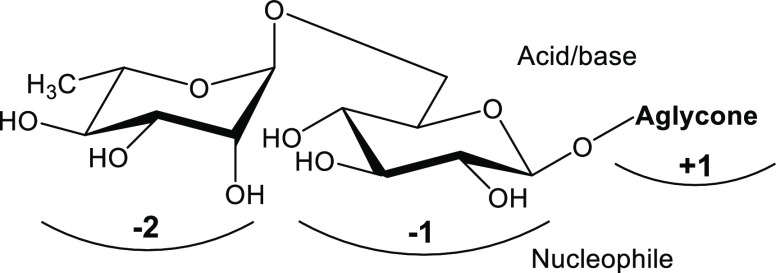
Nomenclature describing the binding subsites of flavonoid-accepting β-glucosidases and β-rutinosidases. The former enzymes possess one negative subsite, whereas the latter enzymes appear to have two negative subsites, which bind the glycone moieties of, e.g., rutin, with the nonreducing end being positioned at the −2 subsite. Cleavage occurs between subsites −1 and +1, where the catalytic acid/base and nucleophile are located. The aglycone binding site is located at the +1 subsite.

**Figure 3:**
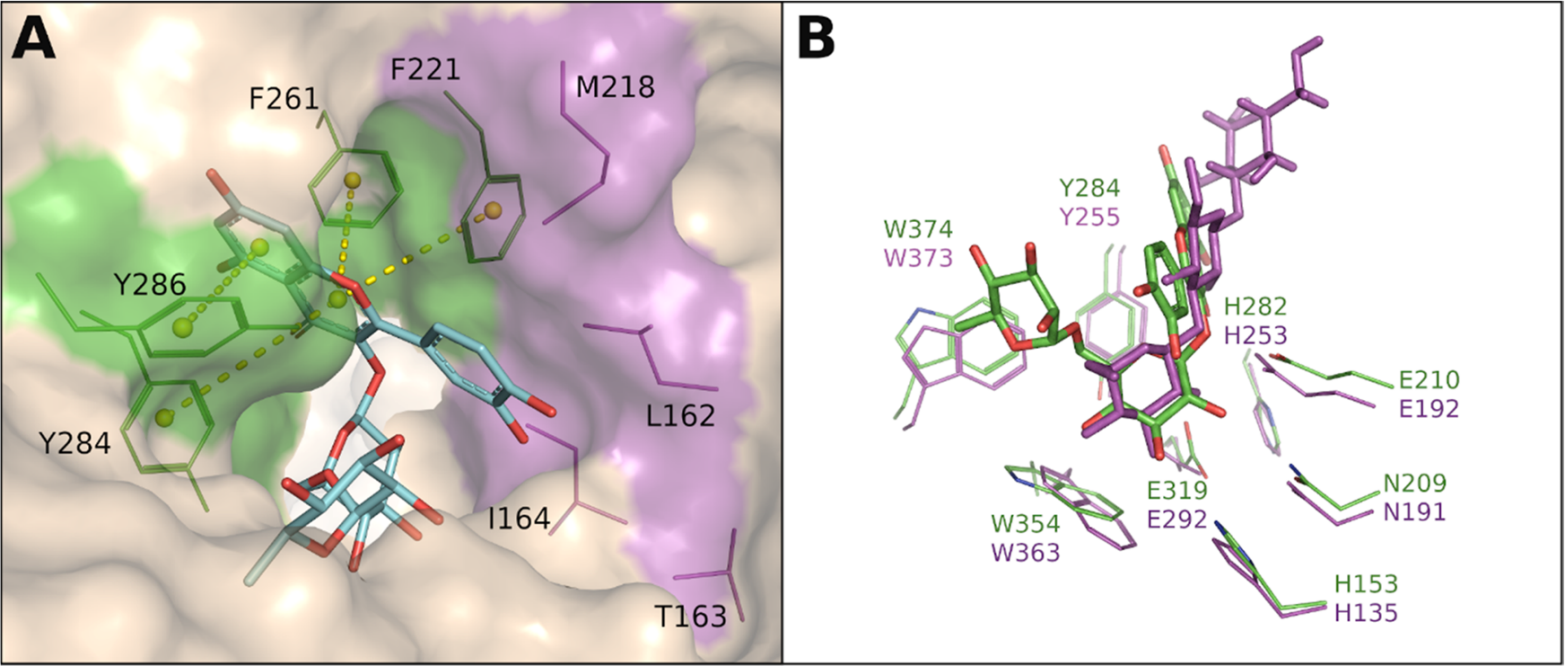
Active site of *An*Rut with bound rutin. (A) Interactions between hydrophobic (magenta) and aromatic residues (green) in the +1 subsite of *An*Rut and docked rutin. The aromatic side chains of F221, F261, F284, and F286 clamp the aglycone moiety by π–π stacking interactions shown as yellow dotted lines.^85,87^ (B) Superposition of common residues in the −1 subsites of *An*Rut (carbon atoms in green, oxygen in red, and nitrogen in blue) and *Ca*Exg (magenta), depicting rutin (green-red) modeled into the active site of *An*Rut, and laminaritriose (β-d-Glc-(1 → 3)-β-d-Glc-(1 → 3)-Glc, magenta) cocrystallized with *Ca*Exg (PDBs 3N9K and 1EQC for the ligand and *Ca*Exg, respectively). The acid/base catalysts Glu210 and Glu192 are also shown.

An important step forward in understanding the structure–function relationships in the β-rutinosidases of the GH5_23 subfamily was the elucidation of the three-dimensional structures in two of them (*Ao*Rut^86^ and *An*Rut^87^). Their high activities toward both β-rutinosides and β-glucosides, such as rutin and isoquercitrin, were traced by their X-ray structures. Molecular docking of rutin in the active site of *An*Rut revealed the mode of substrate accommodation presumably based on hydrophobic and π–π stacking interactions between four aromatic side chains in the +1 subsite and the bound aglycone, with a contribution from polar interactions of the glycone moiety bound in the −1 and −2 subsites (Figure 2). Thus, the +1 subsite in *An*Rut appeared to be specifically designed for flavonoid aglycones with their aromatic and hydrophobic three-ring structures, resulting in sufficient binding strength for both flavonoid-containing rutinosides and β-glucosides (Figure 3A). In these binding poses, the terminal rhamnosyl residue in rutin was located near the entrance to the active site^85,87^ and actually made a negative contribution to the hydrolytic activity of the enzyme, resulting in a 4-fold lower specificity constant *k*_cat_/*K*_M_ for rutin compared with isoquercitrin (77 versus 361 s^–1^ mM^–1^).^87^ Similarly, the best substrate of *Mc*Glc and *Pc*Glc has been found to be isoquercitrin with *k*_cat_/*K*_M_ values of 1.0–1.3 × 10^3^ s^–1^ mM^–1^; that was the reason why these enzymes were termed flavonoid-specific β-glucosidases.^85^ The best diglycoside, narcissin, gave *k*_cat_/*K*_M_ values of 0.3–0.4 × 10^3^ s^–1^ mM^–1^. Large portions of the −1 subsite in *An*Rut have been shown to be structurally highly similar to the corresponding areas of *Ca*Exg. This finding was explained by similar binding interactions at the −1 subsite with the corresponding glucosyl moieties of the respective substrates, i.e., rutin for *An*Rut and laminaritriose for *Ca*Exg (Figure 3B).^87,99^

Interestingly, π–π stacking interactions were also shown to be important in the GH family 1 β-glucosidase Glu1 from maize for the binding of a number of aromatic aglycones in glucosides, such as 4-methylumbelliferyl β-d-glucoside, *p*NP β-d-glucoside, and 2-*O*-β-d-glucopyranosyl-4-hydroxy-7-methoxy-1,4-benzoxaxin-3-one (Figure 1), which was reported to be the natural substrate.^100^ In particular, four aromatic amino acids formed the aglycone binding site of Glu1 and created a hydrophobic surface for aromatic stacking as a key interaction of aromatic aglycone recognition.^101^ A highly hydrophobic aglycone binding pocket with Phe, Val, Trp, Tyr, Met, Leu, and Ile residues lining the walls was also observed in a human cytosolic GH1 β-glucosidase, which hydrolyzed certain flavonoid glucosides with highest specificity constants (*k*_cat_/*K*_M_) of 49.7, 41.8, and 16.6 s^–1^ mM^–1^ for quercetin 4′-*O*-glucoside, apigenin 7-*O*-β-d-glucoside, and quercimeritrin, respectively.^102^ The enzyme is present mainly in the liver, and its role is thought to be the detoxification of certain plant glycosides. *p*NP-containing β-d-fucopyranoside, α-l-arabinofuranoside and β-d-galactopyranoside have also been accepted as substrates. Modeling of enzyme–substrate interactions with quercetin 4′-*O*-glucoside confirmed the involvement of hydrophobic residues in the aglycone binding and the probable absence of hydrogen bonds between the bound flavonol moiety and the protein. In agreement with the determined activity data of the enzyme, molecular docking of flavonoid-3-glucosides such as isoquercitrin was not successful because the mutual positions of the glycone and aglycone binding sites were not compatible with the substrate structure. In a previous work, the enzyme had been shown to prefer β-d-glucosides with planar and hydrophobic aglycones over alkyl β-d-glucosides as substrates.^103^

The importance of the aromatic aglycone for substrate binding and catalysis, deduced from experiments on glucose inhibition of *An*Rut and *Mc*Glc-mediated conversions of rutin, was also stressed for the maize β-glucosidase Glu1.^101,104^ The observed weak inhibition of the hydrolysis reactions by β-d-glucose was found to be consistent with the notion that the glucose moiety alone does not provide enough binding energy for efficient substrate binding and conversion.

**Figure 4:**
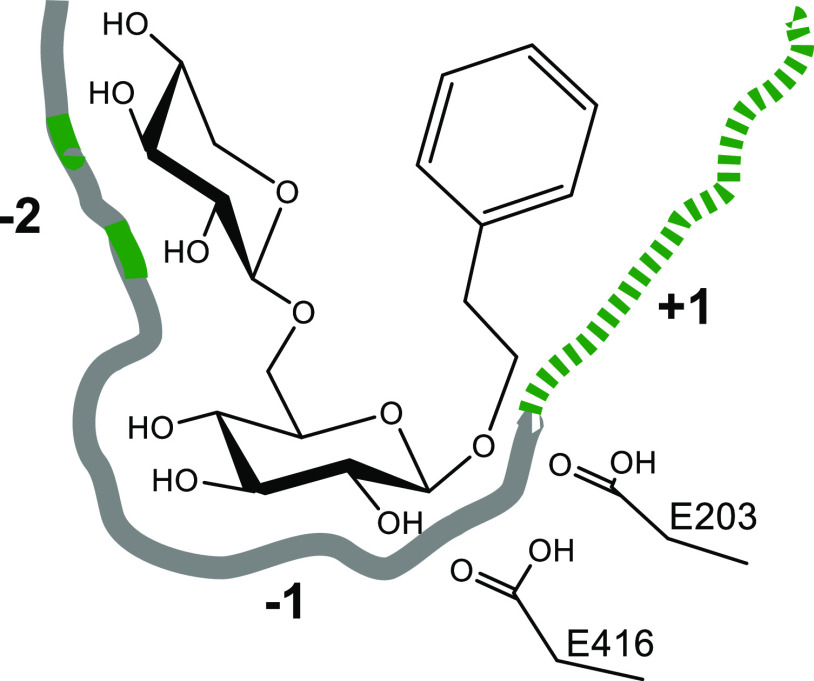
Schematic representation of the substrate binding pose in the active site of PD. Areas that hold the substrate with hydrogen bonds are marked in gray. Active site areas with hydrophobic interactions are highlighted in green. The β-1,6-Xyl-β-Glc moiety of the substrate 2-phenylethyl β-primeveroside is recognized by subsites −2 and −1 and fixed by hydrogen bonds and a few hydrophobic interactions. Subsite +1 is spacious and its interactions with the aglycone have been considered far less important for substrate binding.^105^ It binds the various apolar aglycones mainly nonspecifically through hydrophobic contacts with hydrophobic residues (green dashed line) and the hydrophobic plane of the β-1,6-Xyl pyranose ring. The catalytic nucleophile (E416) and acid/base (E203) are also shown.

As an example of a somewhat dissimilar substrate binding mode to the one of, e.g., *An*Rut, the substrate binding interactions of PD are illustrated in Figure 4, which have been found to result in a combination of strict glycone selectivity and loose aglycone specificity.^105^ PD hydrolyzes the heterosidic linkage between the aglycone and the primeverosyl moiety, releasing primeverose (6-*O*-β-d-xylopyranosyl-β-d-glucopyranose) and a volatile aglycone compound, such as 2-phenylethanol, benzyl alcohol, linalool, or geraniol. The substrate binding site is characterized by a deep funnel-shaped pocket with the aglycone positioned between the pyranose ring of the β-1,6-Xyl moiety in subsite −2 and mostly hydrophobic amino acids in subsite +1 (Figure 4).

**Figure 5:**
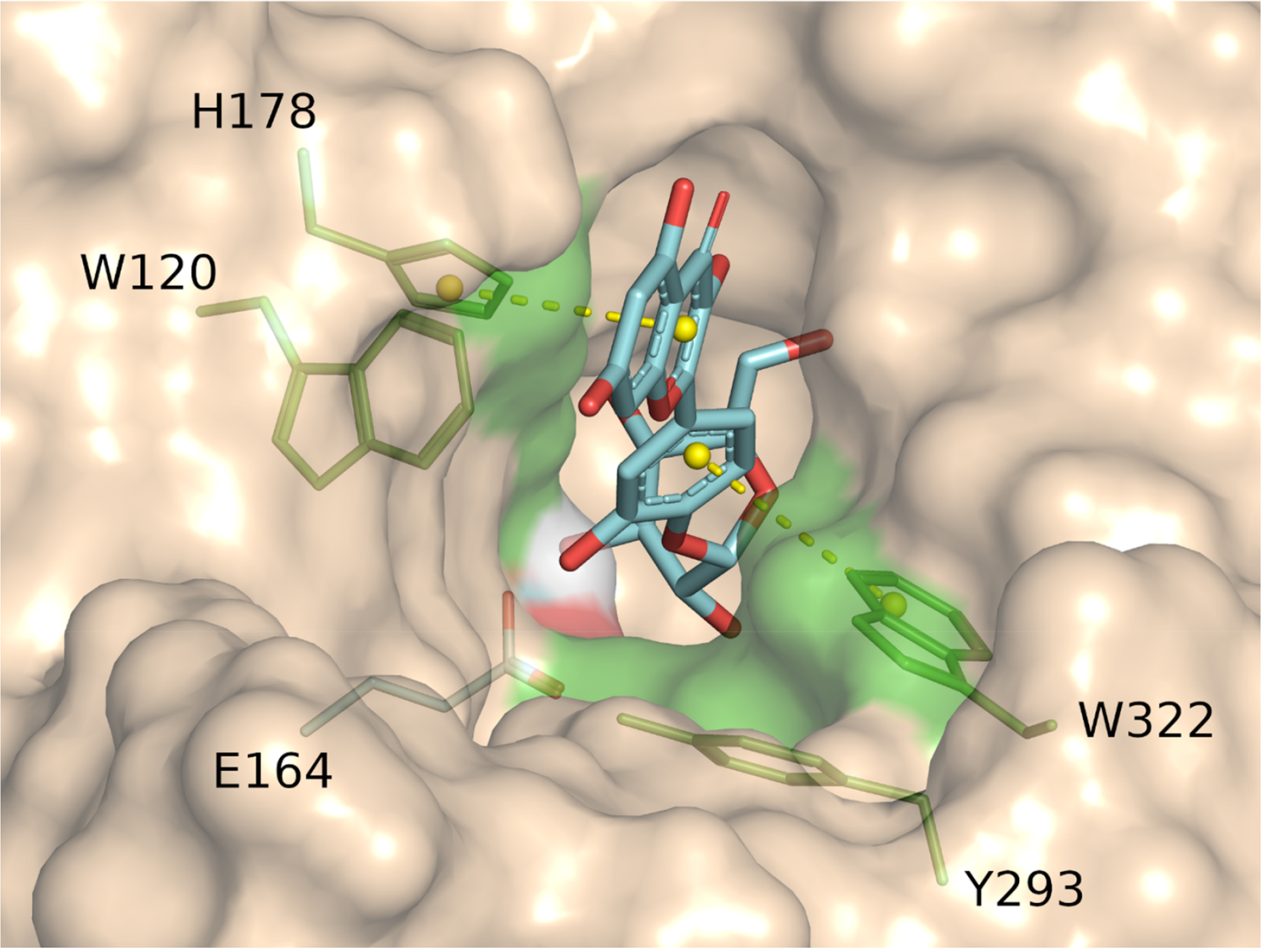
View of the active site of *Tn*Bgl1A with docked quercetin 4′-*O*-glucoside. In the +1 subsite, hydrogen bonds and hydrophobic interactions with aromatic residues (green) were involved in the binding of the substrate;^106^ π–π stacking interactions are shown as yellow dotted lines.

Two mutagenesis studies have been performed with broad-specificity β-glucosidases that deserve closer consideration. Replacement of W322 with alanine in the relatively large and hydrophobic aglycone-binding site of the GH1 β-glucosidase *Tn*Bgl1A (Table 1) resulted in a large decrease in the *k*_cat_ value and catalytic activity for the hydrolysis of *p*NP β-d-glucopyranoside and isoquercitrin. Similarly, the activity toward the other flavonoid 3-*O*-glucosides tested, quercetin 3,4′-di-*O*-β-d-glucopyranoside, kaempferol 3-*O*-β-d-glucopyranoside, and isorhamnetin 3-*O*-β-d-glucopyranoside, was severely affected by the mutation. On the other hand, the ability to hydrolyze the flavonoid 7-*O*-glucosides daidzin, genistin and kaempferol 7-*O*-β-d-glucopyranoside was not affected by the W322A replacement. Molecular docking experiments with the determined X-ray crystal structure of the *Tn*Bgl1A E349G variant indicated the involvement of π–π stacking interactions between the bound flavonoid aglycones in the +1 binding site and W322, resulting in a favorable orientation of quercetin 4′-*O*-β-d-glucoside and quercetin 3,4′-di-*O*-β-d-glucopyranoside for hydrolysis of the glycosidic bond (Figure 5). However, selected amino acid replacements of asparagine residues at positions 220 and 221 resulted in increased specificity constants for the hydrolysis of *p*NP β-d-glucopyranoside and isoquercitrin compared with the wild-type enzyme. This was explained for the N220S mutation by the formation of an additional hydrogen bond with the aglycone of isoquercitrin. N221 was reported not to be involved in any direct contact with the substrate.^106^ The second mutagenesis study dealt with a β-glucosidase from *Neosartorya fischeri*. It revealed the influence of the nonconserved residue N285 in the glycone-binding pocket on the polarity around the general acid/base catalyst E221 and the protonation state of the catalytic nucleophile E430 for the hydrolysis of apigenin 7-*O*-β-d-glucopyranoside, genistein 7-*O*-β-d-glucopyranoside and isoquercitrin. Replacement of N285 with alanine resulted in a total loss of activity, which was explained by an increase in hydrophobicity in the vicinity of E221, resulting in hampered access of catalytic water to the glycosidic bond during the deglycosylation step. In addition, strong perturbations in the p*K*_a_ values of E221 and E430 due to the introduction of the mutation N285R were suspected to be the cause of enzyme inactivation, preventing the proper functioning of the catalytic residues.^107^

## 5 Transglycosylations

Retaining glycosidases have the potential for chemical group transfer reactions and catalyze the transfer of glycosyl groups from donors to acceptors other than water. Although usually in competition with hydrolysis, the group transfer activity of the enzyme leads to the synthesis of new glycosides, with the added acceptor molecule representing the aglycone of the product formed.^108^ Retaining glycosidases that accept flavonoid glycosides allowed the synthesis of a considerable number of novel rutinosylated and glucosylated compounds.^84,85,91,109−113^ These enzymatic products have a high added value because the glycosyl donors rutin and hesperidin are natural and readily available compounds derived from renewable resources, such as plant biomass and biowaste. Enzymatic syntheses of glycosides typically exhibit high to absolute stereo- and regioselectivities and consist of a single reaction as opposed to organic chemistry-based multistep reactions with their obligatory protection and deprotection steps. Therefore, enzymatic conversions can be an extremely attractive and sustainable alternative to synthetic chemistry.

In general, enzyme engineering of retaining glycosidases appears to be necessary to achieve very high transglycosylation yields, which are often rather modest and rarely exceed ∼60–70%, unless difficult-to-identify wild-type transglycosylases can be used.^114^ Glycosyltransferases, which in nature are the key enzymes for the formation of glycosidic bonds, represent another option for glycochemists in the search for suitable syntheses of flavonoid glycosides. However, these enzymes also have their drawbacks that limit their use in biotransformation reactions.^114,115^ Although they are highly efficient and regioselective, they require sugar nucleotides as glycosyl donors, which are very expensive and not readily available. Recycling of sugar nucleotides can help to reduce the economic burden of glycosyltransferase-based processes to some extent. However, this requires the addition of auxiliary enzymes, which makes the reaction setup more complicated. Another disadvantage may be their lower stability and poor heterologous expression. On the other hand, retaining glycosidases are significantly more abundant and cover an extremely broad range of substrate specificities. The disadvantage of retaining glycosidases may be their insufficient ability to catalyze the formation of glycoside bonds compared to the competing hydrolytic reaction. Secondary hydrolysis of the product formed is another common problem that further reduces the transglycosylation yields. Because transglycosylations are kinetically controlled processes, the time dependence of product yields for each reaction should be considered and determined to maximize yields. Moreover, to achieve the highest possible conversions, optimization of reaction conditions, such as catalyst, glycosyl donor and acceptor concentrations, is usually required.^85,116^

### 5.1 β-Rutinosidases

**Figure 6:**
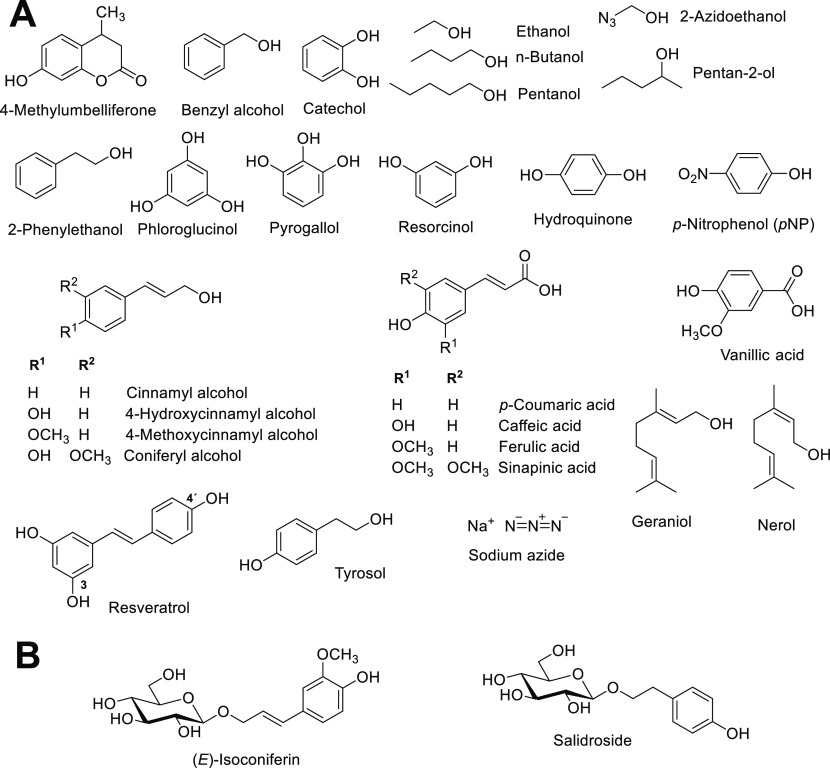
Structures of selected transglycosylation products and sugar acceptors. (A) Acceptors used in transglycosylations using flavonoid-based glycosyl donors and retaining glycosidases. (B) Selected products generated in *An*Rut-mediated transglycosylation reactions with rutin as glycosyl donor and subsequent derhamnosylation using α-l-rhamnosidase.

A broad range of compounds, including, e.g., primary, secondary, and aromatic alcohols (Figure 6), have been reported as acceptors for wild-type β-rutinosidase-mediated transglycosylations; however, tertiary alcohols have been shown to be nonreactive. Looking at the data on *An*Rut-mediated transglycosylations of both small and bulky acceptor molecules, an inverse relationship between transglycosylation activity and acceptor size can be derived as a general trend.^91,110^ For example, very low isolated yields have been reported for the acceptors 4-methylumbelliferone and catechol. For bulkier or phenolic acceptors, higher transglycosylation activities were obtained with β-rutinosidases from *Acremonium* sp. or Tartary buckwheat.^84,112^

Transglycosylations with phenolic acids as acceptors were described with a purified rutinosidase from Tartary buckwheat seeds using 10 mM rutin as a rutinose donor.^111^ The best transglycosylation results were observed for vanillic acid and ferulic acid, generating the corresponding rutinosides in yields of 70% and 45%, respectively. On the other hand, sinapinic acid was the poorest acceptor; however, its rutinoside was the most effective against feline calicivirus compared with the nonglycosylated parent acids and the other rutinosides tested.

**Figure 7:**
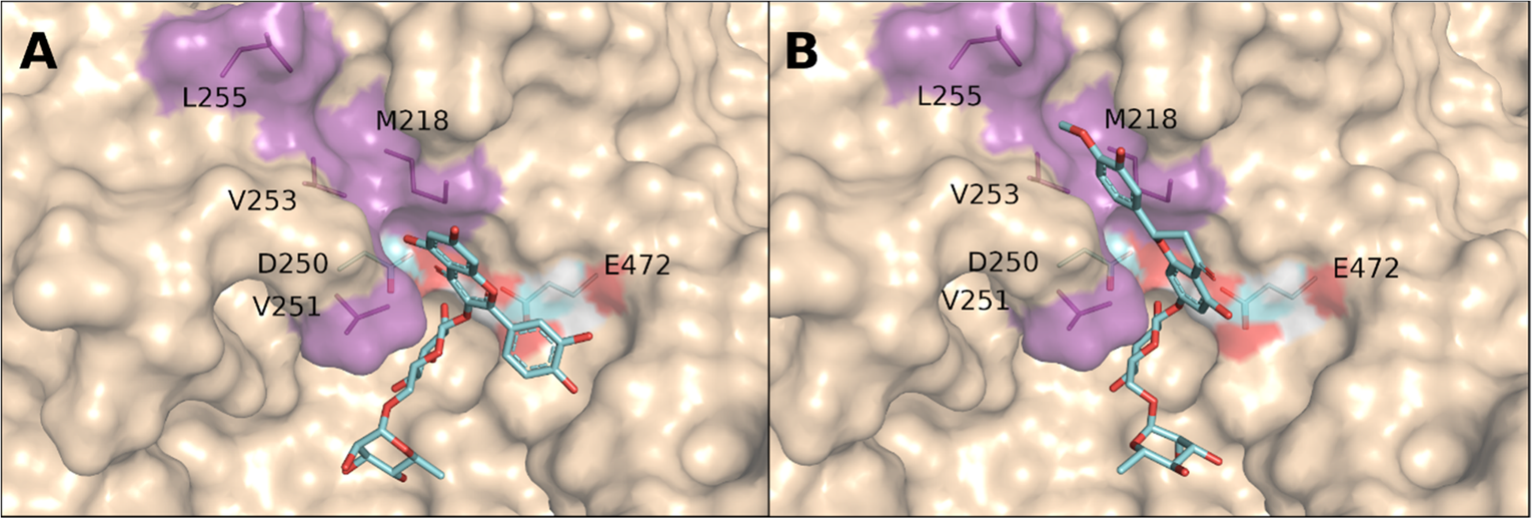
Active site of αRβG II with bound rutin and hesperidin as good substrates. Bound rutin (A) or hesperidin (B) were modeled into the active site, highlighting the hydrophobic residues in the +1 subsite (magenta) that participated in the binding. The presumable catalytic nucleophile Asp250 and acid/base catalyst Glu472 are also depicted.^88^ The structure of αRβG II was obtained by homology modeling using MODELER^153^ and the PDB 4I8D. Docking was performed using Autodock4.^154^

A saprophytic fungal species isolated from soil samples showed deglycosylating activity toward hesperidin in the absence of detectable extracellular α-rhamnosidase and β-glucosidase activities.^117^ The strain was later reidentified as *Acremonium* sp.^118^ Further experiments indicated the presence of an additional rutin-hydrolyzing enzyme in the culture medium when *Acremonium* sp. was growing.^88^ Using the peptide sequences from tryptic digests, both enzyme-encoding sequences were identified in the genome sequence of *Acremonium* sp. The recombinant rutinosidases were termed αRβG I and αRβG II. The diglycosidase activity of αRβG I was restricted to 7-*O*-β-rutinosides such as hesperidin, whereas αRβG II accepted both 7-*O*-β-rutinosides and 3-*O*-β-rutinosides as substrates (Figure 7). In a phylogenetic analysis, αRβG I clustered with other known diglycosidases within GH5_23; on the other hand, the αRβG II-encoding sequence was found in the GH_3 cluster with a β-glucosidase as the closest characterized protein. αRβG I proved to be a valuable catalyst for hesperidin-based transglycosylations. For example, the synthesis of the aroma precursors geranyl and neryl rutinosides was achieved in a biphasic reaction system (10 mL reaction volume) using 1.8 mM hesperidin and 10% (v/v) of the acceptor compound with αRβG I as catalyst.^113^ Using 2-phenylethanol as acceptor, 2-phenethyl rutinoside was synthesized, and the authors reported 80% conversion with no hydrolysis of the synthesized product. Using the same catalyst, but immobilized on chitosan composites, 4-methylumbelliferyl rutinoside was prepared with a yield of 16% in a stirred reactor in the presence of 1.8 mM hesperidin as sugar donor, 1.8 mM acceptor, and 2% (v/v) dimethyl sulfoxide as cosolvent in a volume of 60 mL.^116^ Interestingly, reactions in the presence of 10% (v/v) dimethyl sulfoxide exhibited much higher transglycosylation yields 3 h after the initiation of the reaction. These findings were explained by the better solubility of the substrate at higher proportions of the cosolvent, resulting in better substrate availability for the enzyme. Later, using the same enzyme, various monorutinosylated phenolic compounds were synthesized in transglycosylation reactions using αRβG I with decreasing isolated yields of 38–13% in the order of hydroquinone, catechol, resorcinol, pyrogallol, and phloroglucinol.^112^ In these reactions, a direct relationship was found between the p*K*_a_ value of the phenolic acceptor and the transglycosylation yield. This is in contrast to an expected inverse relationship between the p*K*_a_ value of the acceptor and the transglycosylation yield because hydroxyl groups with lower p*K*_a_ values should be better nucleophiles and thus better competitors for water in attacking the glycosyl–enzyme intermediate. Therefore, factors other than acceptor nucleophilicity came into play with αRβG I-based transglycosylations, such as differential accommodation of phenolic acceptors in the active site of the enzyme.

The first transglycosylations with *An*Rut were published using ethanol, *n*-butanol, and the secondary alcohol pentan-2-ol as acceptors in the presence of rutin as a glycosyl donor and resulted in the formation of the corresponding rutinosides in isolated yields of 46–38%.^91^ In addition, *An*Rut also accepted phenolic compounds, such as benzyl alcohol and catechol, as acceptors for the synthesis of the corresponding rutinosides, albeit in lower isolated yields of 18–27%. Resveratrol as an acceptor afforded two regioisomers in a 2:1 ratio (C3- and C4′-rutinosides) with the glycosidic bond formed with one of the two phenyl groups.

In a subsequent publication, the synthesis of various natural bioactive glucosides with arylalkyl and arylalkenyl aglycones was accomplished in one-pot transglycosylation reactions, adding sequentially *An*Rut and an α-l-rhamnosidase from *Aspergillus terreus* as catalysts.^109^ Using coniferyl alcohol, 4-hydroxycinnamyl alcohol, and cinnamyl alcohol as acceptors, (*E*)-isoconiferin, triandrin, and rosin, respectively, were synthesized in good isolated yields of 68–75% in suspensions of 64 mM rutin and 15% dimethyl sulfoxide. The unconverted rutin and the resulting byproduct quercetin were easily removed from the mixture by centrifugation due to their very low water solubilities. After pH adjustment of the reaction mixture and ethyl acetate extraction, the target β-glucosides were conveniently purified by solid-phase extraction using an Amberlite XAD4 resin and methanol for elution. However, the *An*Rut-based synthesis of salidroside (tyrosol glucoside) and rhamnosylated salidroside resulted in the formation of two regioisomers for both products, requiring an additional purification step. In this regard, the strictly regioselective rutinosidase from dried flower buds of *Sophora japonica*, which exclusively rutinosylated the nonphenolic hydroxyl of tyrosol, proved to be a better option.^82^ Here, the preparative reaction mixtures included a 6-fold excess of acceptor over rutin (49 mM) and 1 g of methanol-treated dried flower buds in a volume of 50 mL.

The full potential of *An*Rut was unleashed with the discovery that it also accepts isoquercitrin as substrate.^87,110^ Thus, with *An*Rut as the sole catalyst, rutinosylated and glucosylated transglycosylation products have been shown to be accessible by using rutin and isoquercitrin, respectively. The synthesis of, for example, pentyl and 2-azidoethyl β-d-glucopyranosides and the corresponding rutinosides was accomplished in the absence of cosolvents using enriched *An*Rut with a 3–6-fold excess of acceptor over rutin (3–6 g) or isoquercitrin (0.5–1.0 g). Reported isolated yields were only moderate: 11–16% for the aliphatic alcohols and <10% for aromatic acceptors, such as catechol or 4-methylumbelliferone. The reaction products were purified by silica gel chromatography after the generated quercetin and the remaining undissolved rutin or isoquercitrin were removed from the reaction mixture by centrifugation and filtration. No product hydrolysis was observed for the aliphatic glycoconjugates, indicating the importance of flavonoid-like aromatic aglycone moieties for good substrate binding and catalysis by *An*Rut. Similarly, the absence of significant hydrolysis of the transglyosylation product 2-phenylethyl rutinoside was reported when using the GH5_23 subfamily enzymes αRβG I, *Mc*Glc, or *Pc*Glc as catalysts.^85,113^

The remarkable formation of rutinosyl esters was described for *An*Rut-catalyzed transglycosylations with rutin as the glycosyl donor and specific acceptors, derivatives of hydroxyphenyl propenoic acid, e.g., (*E*)-ferulic, (*E*)-caffeic, and (*E*)-*p*-coumaric acids. The reaction products were identified to be mixtures of phenolic rutinosides, comprising (*i*) common *O*-glycosides involving the phenolic hydroxyl of the acceptor and (*ii*) glycosyl esters in which the rutinosyl moiety was linked via the carboxyl group of the acceptor.^119^ The data suggest that the formation of glycosyl esters is limited to acceptors with aromatic conjugated systems.

In our search for the most economical conditions for transglycosylations using rutin as a glycosyl donor, we examined two fundamentally different approaches, reactions in solution or in suspension.^85^ Two rutin-hydrolyzing members of the GH5_23 subfamily, *Mc*Glc and *Pc*Glc, were compared for their abilities to form 2-phenylethyl rutinoside in optimized homogeneous and heterogeneous transglycosylation reactions in the presence of 100 and 300 mM rutin, respectively. The comparison showed that under homogeneous reaction conditions in the presence of 25% dimethyl sulfoxide and fully dissolved rutin, double product yields (49% for *Pc*Glc as catalyst) and much better process performance data, turnover frequency, catalyst productivity, and space-time yield, were obtained. Obviously, the high tolerance of *Mc*Glc and *Pc*Glc to dimethyl sulfoxide proved to be a prerequisite for efficient transglycosylations in dimethyl sulfoxide-based reaction mixtures. The possible interference of dimethyl sulfoxide in the reaction mixture with product workup should be mentioned here, although it should be noted that dimethyl sulfoxide can be efficiently removed using solid-state extraction.^120^

Interestingly, wild-type GH 5–23 β-glycosidases, such as *An*Rut, *Mc*Glc, and *Pc*Glc, have been shown to efficiently use inorganic azide as an acceptor in the presence of water for the synthesis of rutinosyl β-azide.^89^ These were not the only wild-type β-glycosidases that could use sodium azide as an external nucleophile: a GH1 β-glycosidase from *Sulfolobus solfataricus* produced β-glucosyl azide in the presence of *p*NP β-d-glucoside as an activated sugar substrate.^121^ Importantly, azido-functionalized carbohydrates can be used as versatile building blocks for the synthesis of glycoconjugates and glycopolymers via the so-called “click-chemistry” method.^122^ Furthemore, glycosyl azides can be an alternative to *p*NP glycoside donors in transglycosylation reactions for the synthesis of disaccharides, as demonstrated with a β-glucosidase, a β-galactosidase, and an α-mannosidase.^123^

### 5.2 Other Glycosidases

Four galactosides derived from myricitrin (myricetin 3-*O*-α-l-rhamnopyranoside), a flavonol glycoside found in, for example, bayberry, were isolated from transglycosylation reactions using lactose as galactosyl donor, myricitrin as acceptor, and a commercial β-galactosidase from *Bacillus circulans*.^124^ The reaction products were isolated in three chromatographic steps and subsequently identified by ESI-MS and NMR spectroscopy as myricitrin decorated with chains of up to three galactosyl residues linked by β-(1 → 3) and β-(1 → 4) bonds. In all cases, the galactosyl moiety was connected to the rhamnosyl residue by an β-(1 → 2) bond. As expected, the water solubility increased dramatically for the galactosylated compounds.

An extracellular β-fructosidase from a dimethyl sulfoxide-tolerant *Arthrobacter nicotianae* strain catalyzed the efficient synthesis of a mixture of fructosylated transglycosylation products using puerarin (daidzein 8-*C*-β-d-glucoside), an isoflavone found in, e.g., the root of *Pueraria*, as an acceptor and sucrose as a fructosyl donor.^125^ The product ratios depended on the solvent system; in the presence of 20–25% dimethyl sulfoxide, which allowed high acceptor concentrations in the reaction mixture, only β-d-mono- and β-d-difructofuranosyl-(2 → 6)-puerarin (connecting the fructosyl moieties with the glucosyl moiety) were formed in excellent yields of 91%. Virtually no secondary hydrolysis of the product was observed.

Finally, (+)-catechin glucoside was formed in one of the very few β-glucosidase-mediated transglycosylation reactions using a bacterial β-glucosidase as catalyst in the presence of *p*NP β-d-glucopyranoside as glycosyl donor; however, the yields were not reported.^126^

### 5.3 Engineered Glycoside Hydrolases

As shown below, engineered active-site mutants of glycosidases, including glycosynthases, thioglycoligases, and *O*-glycoligases, were successfully used in flavonoid-based transglycosylations. The reactions resulted in the formation of flavonoid glycoside products or the depletion of flavonoid-containing glycosyl donors with the synthesis of new glycosides.

**Figure 8:**
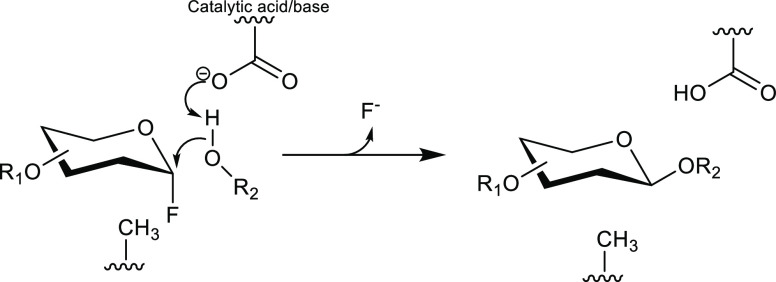
Reaction mechanism of *exo*- and *endo*-β-glycosynthases; R_1_ = additional sugar in the case of *endo*-glycosynthases; R_2_ = acceptor, such as carbohydrate or alcohol. The enzyme lacks the catalytic nucleophile (Glu or Asp), which is replaced by Ala in this example.

**Figure 9:**
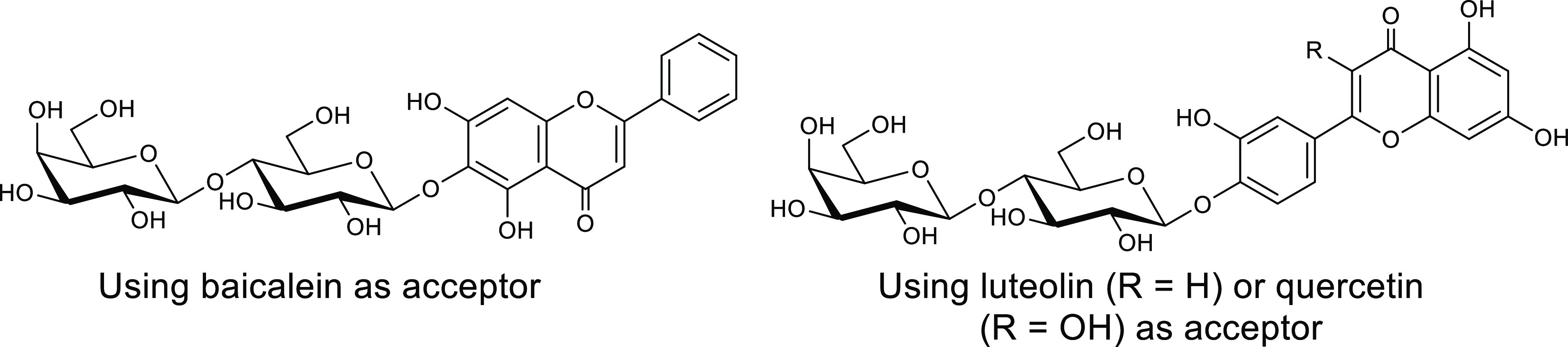
Flavonoid glycoside products obtained in glycosynthase-mediated reactions using the E197S mutant of the Cel7B endocellulase, lactosyl fluoride as diglycosyl donor, and the flavonoid acceptors baicalein, luteolin, and quercetin.

Retaining glycosidase variants lacking the catalytic nucleophile have been shown to be very useful for the synthesis of novel glycosides. These catalytically inactive enzymes, termed glycosynthases, become active in the presence of activated glycosyl donors such as glycosyl fluorides that mimic the covalent glycosyl–enzyme intermediate and therefore have a stereochemistry opposite to that of the naturally occurring substrate (Figure 8).^127−129^ These mutant enzymes eliminate the common problem of product hydrolysis in transglycosylation reactions with wild-type glycosidases, which can lead to low product yields. Using a glycosynthase derived from *Humicola insolens* Cel7B endocellulase, several β-anomers of flavonoid diglycosides were synthesized in the presence of the disaccharide donor α-lactosyl fluoride and flavonoid acceptors, such as baicalein, luteolin, and quercetin (Figure 9).^130^ A similar strategy was used with a glucose-tolerant β-glucosidase from the ascomycete *Talaromyces amestolkiae*, which was converted to a glycosynthase by exchanging the catalytic nucleophile for glycine. α-d-Glycosyl fluoride served as the sugar donor and epigallocatechin gallate as the acceptor, leading to the formation of a mixture of two products composed of β-glucosylated and sophorosylated (i.e., containing a β-1,2-linked disaccharide of glucose) derivatives of epigallocatechin gallate with the sugars attached to the galloyl group.^131^

**Figure 10:**
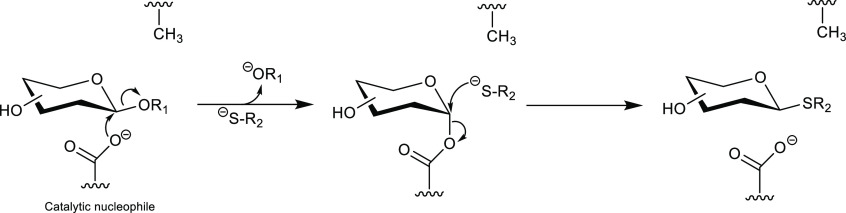
Reaction mechanism of thioglycoligases; R_1_ = leaving group; −SR_2_ = thiol as acceptor.

**Figure 11:**
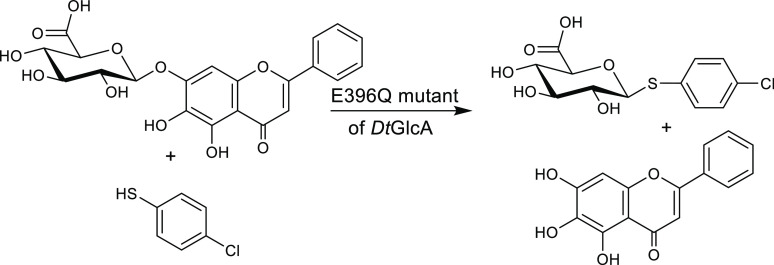
Synthesis of an *S*-glucuronide using the E396Q mutant of the β-d-glucuronidase *Dt*GlcA from *Dictyoglomus thermophilum* as a thioglycoligase, 4-chlorothiophenol as acceptor, and baicalin as a natural glucuronide donor.

Another class of mutants of retaining β-glycosidases are so-called thioglycoligases that lack the general acid/base catalytic residue and have been shown to synthesize nonhydrolyzable *S*-linked disaccharides using a thiol group-containing acceptor and an activated sugar donor of the normal configuration (Figure 10).^132^ Thioglycoligase-mediated reactions are not limited to disaccharide syntheses, as recently demonstrated with a mutant derived from a β-d-glucuronidase from *Dictyoglomus thermophilum*. The constructed thioglycoligase was able to efficiently synthesize an aromatic *S*-glucuronide using 4-chlorothiophenol and baicalin as acceptor and natural glucuronide donor, respectively (Figure 11).^133^ This is another interesting example of a transglycosylation reaction with a naturally occurring carbohydrate donor. The mutant also catalyzed other thioglycoligations, with *p*NP β-d-glucuronic acid as a sugar donor and various aromatic thiol acceptors.

**Figure 12:**
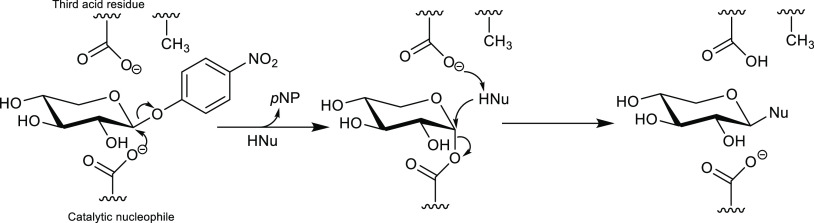
Proposed reaction mechanism of a GH3-based thioglycoligase (E495A mutant of a β-xylosidase) with the third acid residue in the active site facilitating the attack of the incoming nucleophilic acceptor on the anomeric center.^134^ As the sugar donor, *p*NP β-d-xylopyranose is depicted.

**Figure 13:**
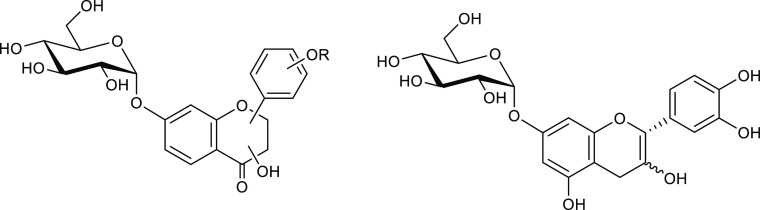
Structures of flavonoid 7-*O*-α-glucoside products generated in transglycosylation reactions using α-d-glucopyranosyl fluoride as a sugar donor and the *O*-α-glycoligase MalA-D416A derived from an α-glucosidase of the thermophilic archeon *Sulfolobus solfataricus*. The left structure represents flavonol, flavanone, flavanonol, and isoflavone glucoside products obtained in these transglycosylations; the right structure represents MalA-D416A-mediated flavanol glucoside products.

A special case has been proposed for thioglycoligases constructed on the basis of members of the GH3 family, most of which were found to have an additional conserved acid residue in their active sites.^134^ This third acid residue was expected to act as an activity-facilitating residue, partially taking over the function of the missing primary acid–base catalytic residue in GH3-based thioglycoligases (Figure 12). The β-xylosidase mutant thus generated proved to be a multiglycoligase and catalyzed the synthesis of a range of *N*-, *S*-, *Se*-, and *O*-xylosides, including *O*-β-xylosylated epigallocatechin gallate, with *p*NP β-d-xylopyranose as the sugar donor. The authors suggested that the remarkably broad acceptor range of the engineered GH3 enzyme was the consequence of (i) easier access of the acceptor to the active site after removal of the primary catalytic acid/base residue, and (ii) the effect of the third acid residue that might help attack the glycosyl-enzyme intermediate without the need for acceptors with strong nucleophilicity. It is also interesting that in this experimental setup the activated glycosyl donor is straightforward to synthesize.^135^ In contrast to β-glycosidase-derived thioglycoligases with their α-glycosyl-enzyme intermediates, α-glycosidases modified at the general acid/base position may react with hydroxyl-containing acceptors, which are less potent nucleophiles than thiols. These findings were explained by the greater reactivity of the β-glycosyl-enzyme intermediate formed in comparison with its α-counterpart in β-glycosidases.^136^ Such α-glycosidase variants, termed *O*-glycoligases, have been employed for the synthesis of various α-glycosides, including 7-*O*-α-glucosylated flavonoids (Figure 13). The compounds were synthesized regioselectively using α-d-glucopyranosyl fluoride as the activated sugar donor, the α-glucosidase variant MalA-D416A from *Sulfolobus solfataricus*, and acceptors, such as quercetin, kaempferol, hesperetin, naringenin, (+)-taxifolin, genistein, and daidzein, which represent flavonol, flavanone, flavanonol, and isoflavone types of flavonoids.^137^ The optimal pH for the mutant-mediated reaction was determined to be 9.0, in contrast to pH 5.0 for the hydrolysis reaction. No formation of transglycosylation products was observed at pH < 6.0, suggesting that enhancing the nucleophilicity by deprotonation of the hydroxyl at position C-7 of the flavonoid acceptor is important for the reaction.

**Figure 14:**
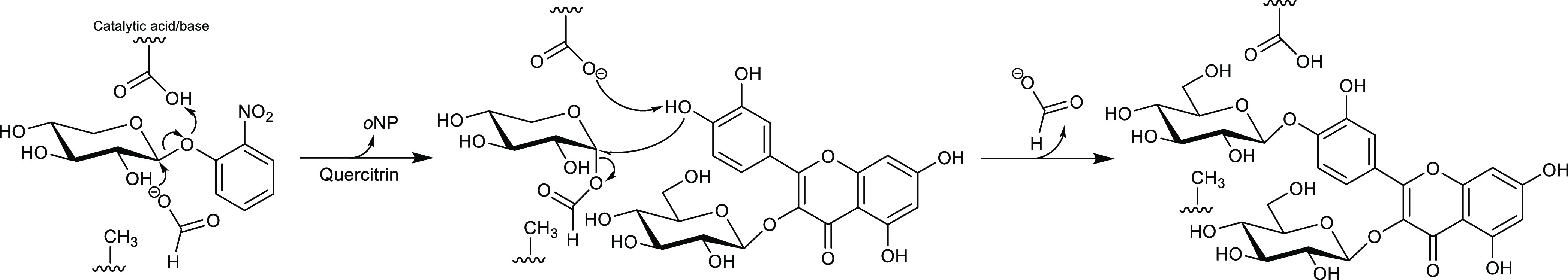
Formation of quercetin 3,4′-di-*O*-β-glucoside by virtue of the glycosynthase activity of *Tn*Bgl1A-E349G in the presence of sodium formate as an external nucleophile using *o*NP β-d-glucopyranoside as the donor and isoquercitrin as acceptor.

Strong external nucleophiles have been used with inactive glycosidase mutants in so-called activity rescue experiments to identify the acid/base catalyst and the catalytic nucleophile in retaining glycosidases.^138^ The lost assistance of the catalytic nucleophile or the general acid/base catalyst, as a consequence of their replacement by catalytically inert residues, was overcome by high concentrations of small nucleophiles, such as formate or inorganic azide, in the reaction mixture with activated glycosides as glycosyl donors (e.g., *p*NP glycosides), restoring the transglycosylation activity of the retaining glycosidases. In general, the anomeric configuration of the glycoside formed was found to be the same as that of the glycosyl donor in acid/base-catalyst mutants, whereas an inverted anomeric configuration of the product was observed in nucleophile mutants. The concept of external nucleophiles and catalytic nucleophile mutants was later exploited for the synthesis of quercetin 3,4′-diglycosides and rutinosyl α-azide. The combination of the catalytic nucleophile mutant of *An*Rut and inorganic azide as an external nucleophile in the presence of the glycosyl donor rutin allowed the facile and efficient preparative synthesis of rutinosyl α-azide, whose chemical synthesis is challenging.^89^ Regarding the synthesis of quercetin 3,4′-diglycosides, three catalytic nucleophile variants of *Tn*Bgl1A were successfully tested for their glycosynthase activities using two different approaches with isoquercitrin as acceptor.^139^ One set of experiments was performed with α-glucosyl fluoride as donor, whereas the other set was performed in the presence of formate anions and *o*NP or *p*NP β-d-glucopyranoside as donor, using the concept of *in situ* generation of a noncovalently bound α-glycosyl formate intermediate (analogous to the covalent α-glycosyl-enzyme intermediate of retaining glycosidases)^121^ that served to synthesize β-linked quercetin glycosides after nucleophilic attack of an activated carbohydrate acceptor molecule. The formate anions present merely mimicked the function of the removed carboxylate group of the catalytic nucleophile and were not incorporated into the reaction product (Figure 14). Both approaches yielded quercetin 3,4′-di-*O*-β-glucoside as the major product with the highest yield of 37% determined for the E349G variant and the donor *o*NP β-d-glucopyranoside. No product formation was observed with quercetin as acceptor. Remarkably, the enzymatic reactions were performed in 50% (v/v) methanol. Using molecular docking, the authors identified W322 and Y175 in the aglycone binding site as important residues involved in stacking and/or hydrophobic interactions with the bound quercetin moiety of the acceptor molecule.^139^ In a later project, a functional glycosynthase based on the GH3 β-glucosidase *Tn*Bgl3B from *Thermotoga neapolitana* was constructed by introducing two mutations, focusing on the catalytic nucleophile (D242A) and the −1 subsite (W243F). In contrast to the single mutant D242A, the double mutant was active in the presence of the external nucleophile formate, *p*NP β-d-glucopyranoside as donor and the following acceptors: isoquercitrin, quercetin 3-*O*-β-d-galactoside, or quercetin. In all three cases a quercetin 3,4′-diglycoside was formed in yields of up to 40%.^140^

Glycoside phosphorylases also have the capability to transfer glycosyl moieties to (noncarbohydrate) acceptor molecules.^141^ Like retaining glycosidases, they operate in two distinct catalytic steps via a double displacement mechanism with the formation of a covalent enzyme–glucosyl intermediate that is subsequently attacked by the incoming acceptor molecule, resulting in the release of a glucoside. Very interesting biocatalysts are sucrose phosphorylases, which catalyze the reversible phosphorolysis of sucrose to d-fructose and α-d-glucose 1-phosphate because low-cost sucrose can participate in the transglycosylation reaction as a very efficient glucosyl donor. For example, the sucrose phosphorylase variant R134A from *Thermoanaerobacterium thermosaccharolyticum* enabled the glucosylation of quercetin, catechin, or epicatechin in the presence of sucrose.^142^ The R134A mutation ensured better access of bulky flavonoid acceptor molecules to the enlarged active site. In the case of (+)-catechin and (−)-epicatechin, good isolated yields of 31% and 58% were reported for the corresponding 3′-*O*-α-d-glucopyranoside products. The glycone of the latter product was subsequently elongated using α-d-glucose 1-phosphate as a donor in the presence of a cellodextrin phosphorylase, giving the epicatechin-based cellobioside in low yield. Another example is the Q345F variant of a sucrose phosphorylase from *Bifidobacterium adolescentis*, which has been shown to efficiently glucosylate selected flavonoids in contrast to the wild-type enzyme, which strongly preferred hydrolysis.^143−145^ The various transfer reactions yielded naringenin 7-*O*-α-d-glucoside, fisetin 3′-*O*-α-d-glucoside with an uncharacterized product, a mixture of 3′-*O*-α-d-monoglucosylated, and 3′,5-*O*-α-d-diglucosylated (+)-catechin, or three (−)-epicatechin or quercetin glucosides. With respect to hydrolysis, the mutant enzyme showed an ∼10-fold lower specific activity toward sucrose compared with the wild-type enzyme. Based on the crystal structures of the wild-type enzyme and its Q345F variant in the absence of a ligand and in complex with glucose or resveratrol 3-α-d-glucoside, the authors were able to deduce the structural reasons for the greatly enhanced glucosylation activity. The X-ray data revealed that the mutation induced extensive structural changes of a very dynamic nature, including additional space in the active site, modified and reduced hydrogen bond interactions between the enzyme and the donor molecule, and the formation of an aromatic surface for hydrophobic and π–π interactions with the flavonoid acceptor. An additional mutation, P134D, improved the regioselectivity of the enzyme resulting in 82% of the product mixture formed as (+)-catechin 3′-*O*-α-d-glucoside.^146^

Naturally occurring transglycosylases are special glycoside hydrolases in that they preferentially or exclusively catalyze transglycosylations at the expense of hydrolysis.^147^ They appear to follow the same reaction mechanism of most retaining glycoside hydrolases, and they served as inspiration for the engineering of hydrolytic glycoside hydrolases with the aim of improving their transglycosylation activities.^114^ In this context, rules for improving the ratio of hydrolysis and transglycosylation in glycoside hydrolases by enzyme engineering were established.^114^ The reverse approach was followed with the GH1 transglycosylase Os9BGlu31 from rice. The wild-type enzyme was shown to catalyze the synthesis of kaempferol and apigenin 7-*O*-glucosides using *p*NP β-d-glucoside as donor.^148,149^ In subsequent publications, the authors introduced a series of mutations at position 243 in the active site cleft of Os9BGlu31 and analyzed the enzyme variants in terms of their activities with selected phenolic acids and flavonoids. It was found that the W243N variant exhibited higher hydrolysis rates compared with the wild-type Os9BGlu31 enzyme and, in contrast to the wild-type enzyme, formed several mono- and bis-*O*-glucoconjugates with kaempferol as the acceptor and *p*NP β-d-glucoside as glucosyl donor.^150^ The introduction of a hydrophilic residue into the putative water-binding site resulted in an even higher overall ratio of hydrolysis to transglycosylation when the double variant L183Q/W243Q was generated,^149^ supporting the notion that hydrophilic residues in the binding site for catalytic water tend to increase hydrolytic activity due to the facilitated presence of water.

## 6 Outlook

Flavonoids are very abundant phenolic compounds in all types of food plants, and their importance in human nutrition and health is well recognized. In nature, flavonoids are associated with the action of specific glycoside hydrolases, as they often occur as glycoconjugates that usually have to be hydrolyzed to be absorbed and exert biological activities. In biotechnology, retaining glycosidases with their hydrolytic and transglycosylating abilities, either as wild-type or engineered enzymes, are used for the hydrolysis or synthesis of flavonoid glycosides and therefore represent a valuable biocatalytic platform. Because flavonoid glycosides are involved in all kinds of biological activities, with many of them presumably still unknown, their importance is expected to increase in the near future. Simple and high-yielding syntheses of flavonoid glycosides, which serve as standards, probes, and samples in biochemical and biomedical experiments and applications, are needed. In addition, specific flavonoid glycosides, such as rutin, hesperidin, and naringin, can be used as readily available, inexpensive and sustainable glycosyl donors from biomass-based feedstocks for glycosidase-mediated synthesis of natural or novel glycosides. Furthermore, enzymatic regioselective modifications of flavonoids, e.g., by hydroxylation^151^ or the conversion of readily available hesperetin to diosmetin using flavone/flavonol synthases,^152^ are further emerging routes toward the full exploitation of the many untapped flavonoid-linked enzyme activities for future successful, viable and sustainable processes. All of these may further extend the competitive advantage of enzymatic transglycosylations over the chemical synthesis approach. From a perspective of green chemistry and sustainability, the ultimate goal would be to produce flavonoid or other glycosides by transglycosylation in very high yields using convenient retaining glycosidases and low-cost glycosyl donors, preferably of natural origin. To achieve this goal, suitable naturally occurring transglycosylases need to be identified, and engineered retaining glycosidases with high transglycosylation activities need to be developed.

## Abbreviations

αRβG I/II: β-rutinosidases from *Acremonium* sp. DSM 24697
*An*Rut: β-rutinosidase from *Aspergillus niger* K2 CCIM
*Ao*Rut: β-rutinosidase from *Aspergillus oryzae* RIB40
*Ca*Exg: *exo*-β-(1,3)-glucanase from *Candida albicans*
GH: glycoside hydrolase
Glc: d-glucosyl
GlcA: glucuronosyl
*Mc*Glc: β-glucosidase/rutinosidase from *Mucor circinelloides* CCF 2598
*Pc*Glc: β-glucosidase/rutinosidase from *Penicillium chrysogenum* CCF 1269
PD: β-primeverosidase from *Camellia sinensis* (tea plant)
*o*NP: *ortho*-nitrophenyl
*p*NP: *para*-nitrophenyl
TMO: 2,2,5,5-tetramethyloxolane
*Tn*Bgl1A: β-glucosidase from *Thermotoga neapolitana*
Xyl: β-d-xylosyl
